# Overarching view of trends and disparities in malignant neoplasm of the ovary between 1999-2023: a comprehensive CDC WONDER database study

**DOI:** 10.3389/fonc.2025.1691932

**Published:** 2025-11-04

**Authors:** Jaikin Patel, Daniel Murillo Armenta, Olivia Foley, Abubakar Tauseef

**Affiliations:** ^1^ Creighton University School of Medicine, Omaha, NE, United States; ^2^ Department of Internal Medicine, Creighton University School of Medicine, Omaha, NE, United States

**Keywords:** ovarian cancer, ovarian neoplasm, mortality, health disparities, CDC WONDER database

## Abstract

**Background:**

Ovarian cancer contributes significantly to the morbidity and mortality rates for women worldwide. As observed with other types of cancer, health disparities disproportionately affect ovarian cancer incidence rates and outcomes, especially in African American and older women. However, the trends in ovarian cancer mortality rates up until 2023 with regard to various demographic identifiers have not been fully elucidated, which this study aims to rectify.

**Methods:**

Mortality trends due to malignant neoplasms of the ovary in individuals 25 and older in the US from 1999 to 2023 were analyzed using the Centers for Disease Control Wide Ranging Online Data for Epidemiological Research (CDC WONDER) database. Trends in age-adjusted mortality rate (AAMR) were analyzed on the basis of race, 10-year age-group, region and urban/rural designation.

**Results:**

Between 1999 and 2023, the AAMR related to malignant neoplasms of the ovary fell from 14.62 in 1999 to 9.52 in 2023. All races analyzed saw a decrease in overall mortality related to malignant neoplasms of the ovary, with the largest decrease being observed in White patients (AAPC: -1.78). Regionally, the Northeast (AAPC: -1.95), Midwest (AAPC: -1.99), South (AAPC: -1.72), and West (AAPC: -1.73) regions of the United States (US) all saw reduced ovarian neoplasm mortality rates. Similarly, rates also decreased in urban (AAPC: -1.83) and rural (AAPC: -1.75) localities, as well as in each ten-year age category analyzed, with the largest decrease seen in the 55–64 years old category (AAPC: -2.15). States such as Delaware, South Carolina, and Idaho experienced some of the largest decreases in AAMR, whereas the District of Columbia saw an increase in AAMR during this period.

**Conclusions:**

Over the last twenty-years, mortality rates for malignant neoplasms of the ovary have declined, with the largest decreases being seen in White patients, those residing in the Midwest, urban locality, and women between 55–64 years olds. While mortality rates have declined, health disparities still continue to negatively affect ovarian cancer outcomes, and more research is needed to improve accessibility, availability, and affordability of care for patients.

## Introduction

Ovarian cancer is a significant cause of morbidity and mortality for women worldwide. Amongst women with cancer, ovarian cancer ranks fifth in terms of mortality - in fact, ovarian cancer may be the deadliest of all gynecological cancers in women (1). Early detection of ovarian cancer remains an important prognostic factor; however, initial symptoms of ovarian cancer are often non-specific, if they are even present at all (back pain, abdominal pain, fatigue, etc.). Thus, early detection of this disease state is difficult. Additionally, routine screening for ovarian cancer is not currently recommended unless the patient has a genetic predisposition or family history of the disease (2).

Like many other cancer subtypes, health disparities appear to impact the incidence of ovarian cancer amongst various racial groups. For example, African American women at all stages of the disease appear to be impacted to a greater extent compared to non-Hispanic White women (3). In fact, African American patients were 17-18% less likely to survive a diagnosis of ovarian cancer compared to White patients (4). Some researchers, such as Chornokur, attribute these disparities to unequal access to care and a lack of standardized treatment regiments (2012). Thus, the purpose of this study is to further analyze trends in ovarian cancer mortality rates from 1999 to 2023 with regards to various demographic identifiers to add to existing efforts to improve the diagnosis, treatment, and outcomes for ovarian cancer patients overall.

## Methods

### Data collection

Mortality trends due to malignant neoplasms of the ovary in individuals 25 and older in the US from 1999 to 2023 were analyzed using the Centers for Disease Control Wide Ranging Online Data for Epidemiological Research (CDC WONDER) database, a publicly available database containing information relevant for public health analysis. Adults aged 25 and over were included in the study, as younger age groups did not have sufficient or reliable ovarian neoplasm-related mortality data. Data was extracted from the CDC WONDER database using the International Classifications of Disease (ICD), 10th revision code C56. Stratification was performed by race, 10-year age-group, region and urban/rural designation. Race was analyzed by separating Hispanic from Non-Hispanic ethnic categories, whereby Non-Hispanic racial categories included American Indian, Asian or Pacific Islander, White, and Black or African American. Regions, as defined by the Census Bureau classification, included South, West, Midwest and Northeast. Data from any group or stratification that was marked unreliable by the CDC WONDER database, indicating that there was an insufficient amount of deaths in that subgroup to perform proper analysis, were excluded from final results of this study.

The Institutional Review Board (IRB) approval was not required for this study. This study was conducted in compliance with the ethical standards of the responsible institution on human subjects as well as with the Helsinki Declaration.

### Data analysis

Age-adjusted mortality rates (AAMRs) were calculated to account for differences in age distribution across populations, enabling accurate comparisons. Furthermore, AAMR were standardized to the 2000 U.S. standard population (5). Mortality trends over the study period were analyzed using the Joinpoint Regression Program (version 5.4.0.0) developed by the National Cancer Institute (6). This tool applied segmented regression modeling to the AAMRs to identify changes in trend, estimating Annual Percent Changes (APCs) and Average Annual Percent Changes (AAPCs). APCs and AAPCs, and their corresponding 95% confidence intervals were calculated for each segment using the Monte Carlo permutation method, which determines the optimal number and location of joinpoints. Statistical significance was assessed using two-sided t-tests, with a threshold of p <0.05 (7). The number of joinpoints utilized for each analysis was selected by the program, utilizing the best-fit model approach. Missing data was excluded from analysis to ensure the most accurate and reliable results. Any data that was deemed insufficient or unreliable by the CDC WONDER database was also excluded. Statistically significant values are indicated with an asterisk in all reported tables and figures.

## Results

### Overall

**Figure 1:**
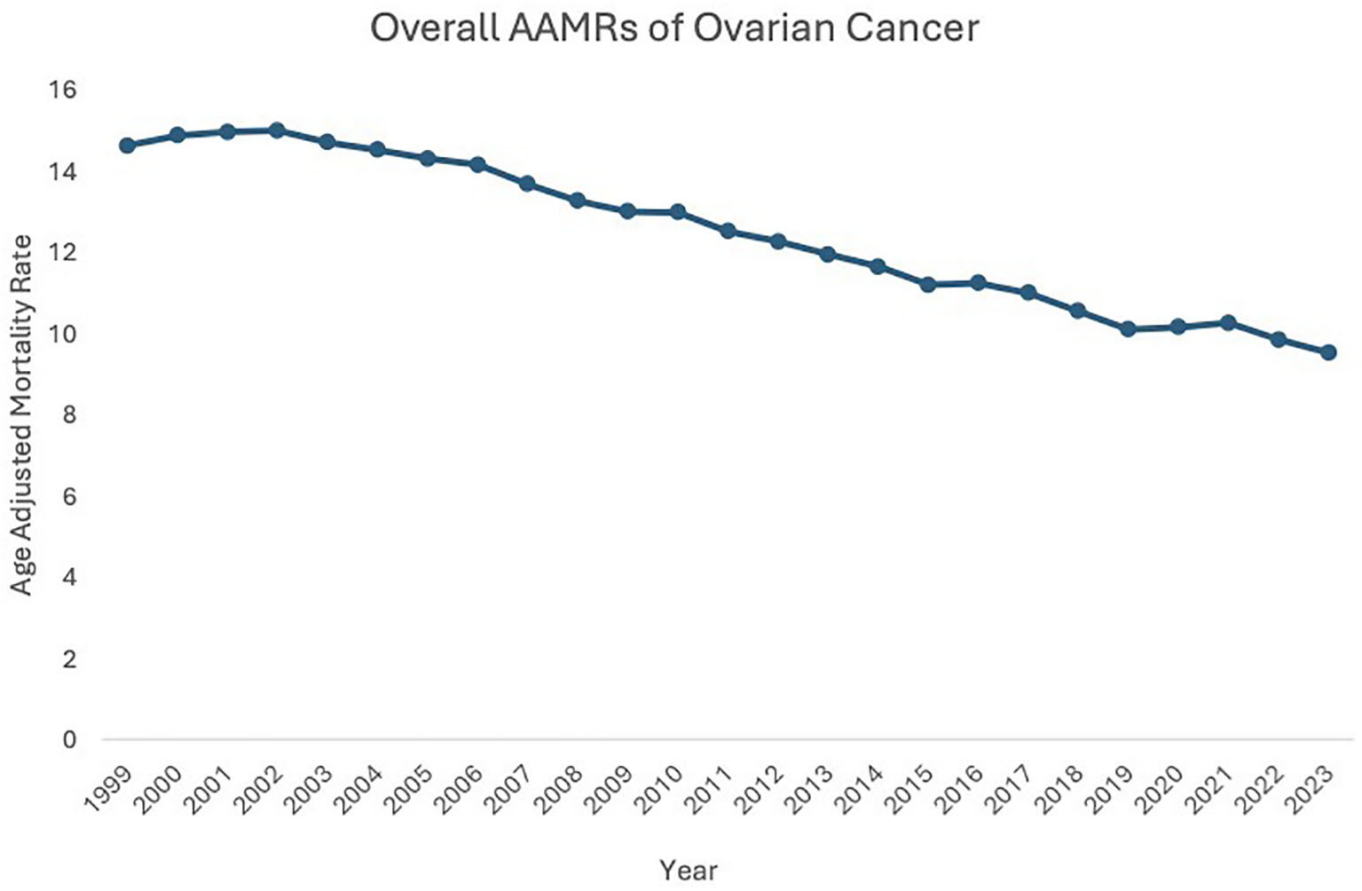
Overall AAMRs of ovarian cancer.

Between 1999 and 2023, the age-adjusted mortality rate (AAMR) related to malignant neoplasms of the ovary fell from 14.62 in 1999 to 9.52 in 2023, highlighting a 1.80% decrease in the Average Annual Percent Change (AAPC) during this period. AAMR initially increased from 1999 to 2003 (APC: 0.30, 95% CI: -0.62 to 1.76), then decreased from 2003 to 2023 (APC: -1.80, 95% CI: -2.32 to -2.13) (**Figure 1**, **Supplementary Figure 1**).

### Race stratified

Between 1999 and 2023, American Indian or Alaska Native (AAPC: -1.19, 95% CI: -2.41 to 0.25), Asian or Pacific Islander (AAPC: -0.75, 95% CI: -1.04 to -0.39), Black or African American (AAPC: -1.37, 95% CI: -1.79 to -1.00), White (AAPC: -1.78, 95% CI: -1.89 to -1.65), and Hispanic (AAPC: -1.23, 95% CI: -1.44 to -1.00) patients all saw a decrease in overall mortality related to malignant neoplasms of the ovary.

**Figure 2:**
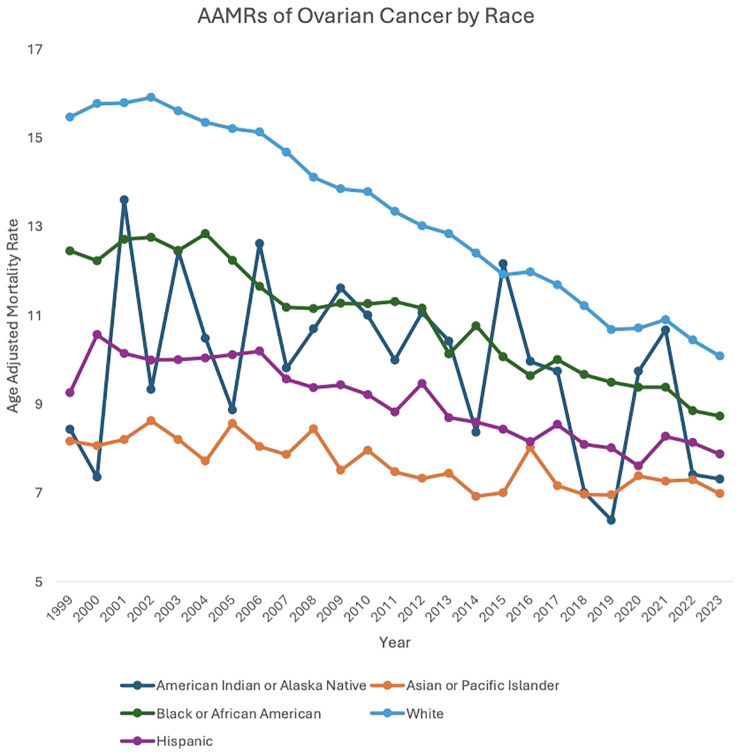
AAMRs of ovarian cancer by race.

For the majority of the study period, White patients experienced a higher AAMR compared to the other races analyzed. Between 1999 and 2003, White patients’ AAMR increased from 15.47 in 1999 to 15.61 in 2003 (APC: 0.53, 95% CI: -0.51 to 2.22) and subsequently decreased from 15.61 in 2003 to 10.08 in 2023 (APC: -2.24, 95% CI: -2.36 to -2.14). Between 1999 and 2002, Black or African American patients’ AAMR increased from 12.45 in 1999 to 12.76 in 2002 (APC: 1.23, 95% CI: -1.55 to 6.82), which then decreased to 8.73 in 2023 (APC: -1.73, 95% CI: -3.74 to -1.53). American Indian or Alaska Native patients’ AAMR decreased from 8.43 in 1999 to 7.31 in 2023 (APC: -1.19, 95% CI: -2.41 to 0.25). Asian or Pacific Islander patients’ AAMR also decreased from 8.16 in 1999 to 6.98 in 2023 (APC: -0.75, 95% CI: -1.04 to -0.39). Hispanic patients’ AAMR decreased from 9.26 in 1999 to 7.87 in 2023 (APC: -1.23, 95% CI: -1.44 to -1.00) (**Figure 2**, **Supplementary Figure 2**, **Supplementary Table 1**).

### Region stratified

Between 1999 and 2023, the Northeast (AAPC: -1.95, 95% CI: -2.11 to -1.76), Midwest (AAPC: -1.99, 95% CI: -2.18 to -1.75), South (AAPC: -1.72, 95% CI: -1.88 to -1.53), and West (AAPC: -1.73, 95% CI: -1.95 to -1.49) regions of the United States (US) all saw decreases in overall malignant neoplasm of the ovary-related mortality.

**Figure 3:**
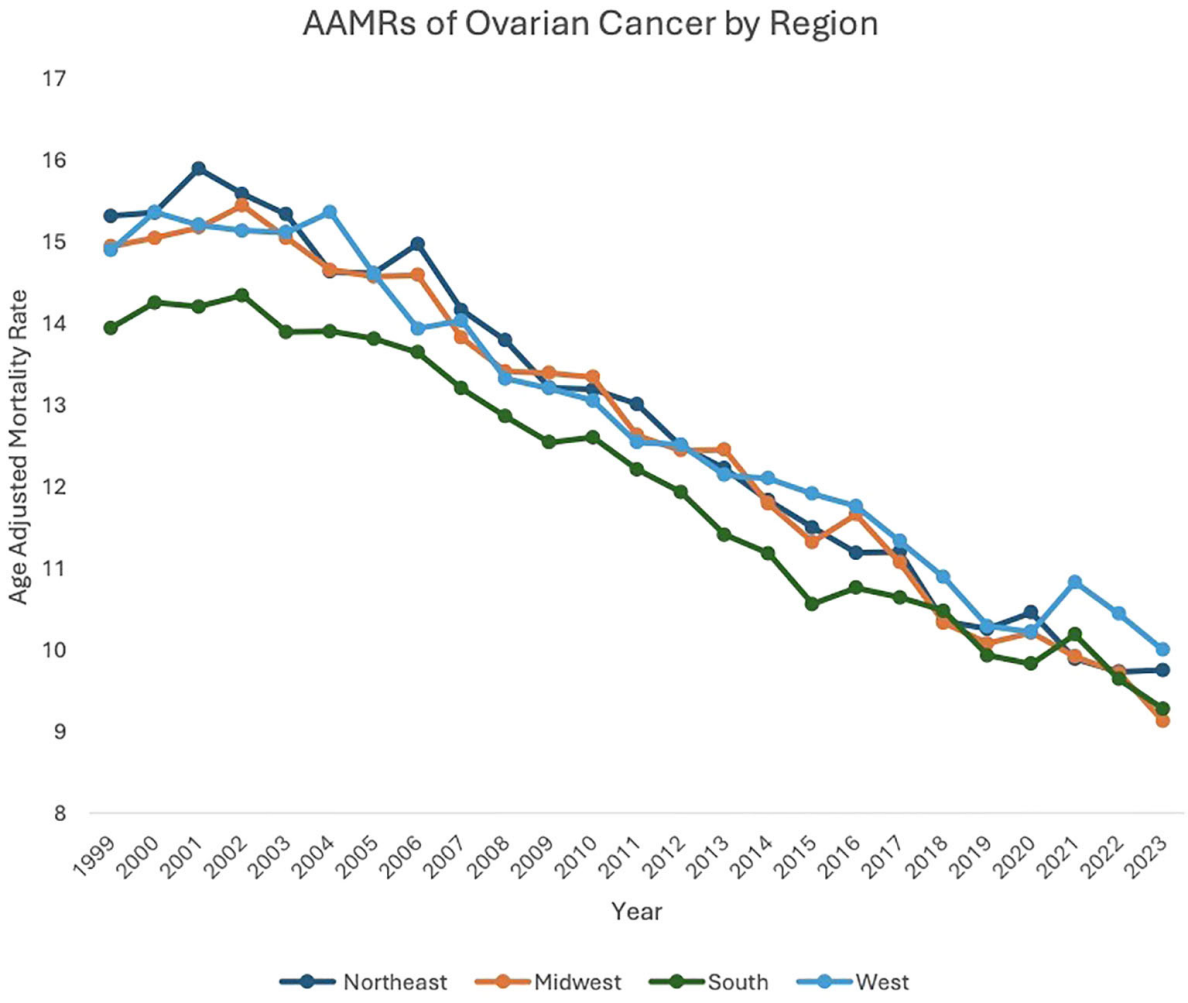
AAMRs of ovarian cancer by region.

Between 1999 and 2002, patients in the Northeast region saw an increase in AAMR, from 15.31 in 1999 to 15.58 in 2002 (APC: 1.11, 95% CI: -0.83 to 4.48), which then decreased to 9.75 in 2023 (APC: -2.38, 95% CI: -2.55 to -2.25). Between 1999 and 2005, Midwest patients saw a decrease in AAMR, from 14.94 in 1999 to 14.57 in 2005 (APC: -0.41, 95% CI: -1.35 to 1.59). From 2005 to 2023, Midwest patients’ AAMR decreased further, from 14.57 in 2005 to 9.13 in 2023 (APC: -2.51, 95% CI: -2.81 to -2.31). Between 1999 and 2004, patients in the South saw a decrease in AAMR, from 13.94 in 1999 to 13.90 in 2004 (APC: -0.15, 95% CI: -1.16 to 2.29). Between 2004 to 2023, patients in the South saw a further decrease in AAMR, to 9.28 in 2023 (APC: -2.13, 95% CI: -2.33 to -1.99). Between 1999 and 2003, patients in the West saw an increase in AAMR, from 14.89 in 1999 to 15.11 in 2003 (APC: 0.04, 95% CI: -1.57 to 3.94). AAMR in the West then decreased to 10.00 in 2023 (APC: -2.08, 95% CI: -2.42 to -1.93) (**Figure 3**, **Supplementary Table 2**, **Supplementary Figure 3**).

### Urban-rural stratification

From 1999 to 2020, both urban (AAPC: -1.83, 95% CI: -1.94 to -1.68) and rural (AAPC: -1.75, 95% CI: -1.75 to -1.32) localities saw a decrease in overall ovarian neoplasm-related mortality.

**Figure 4:**
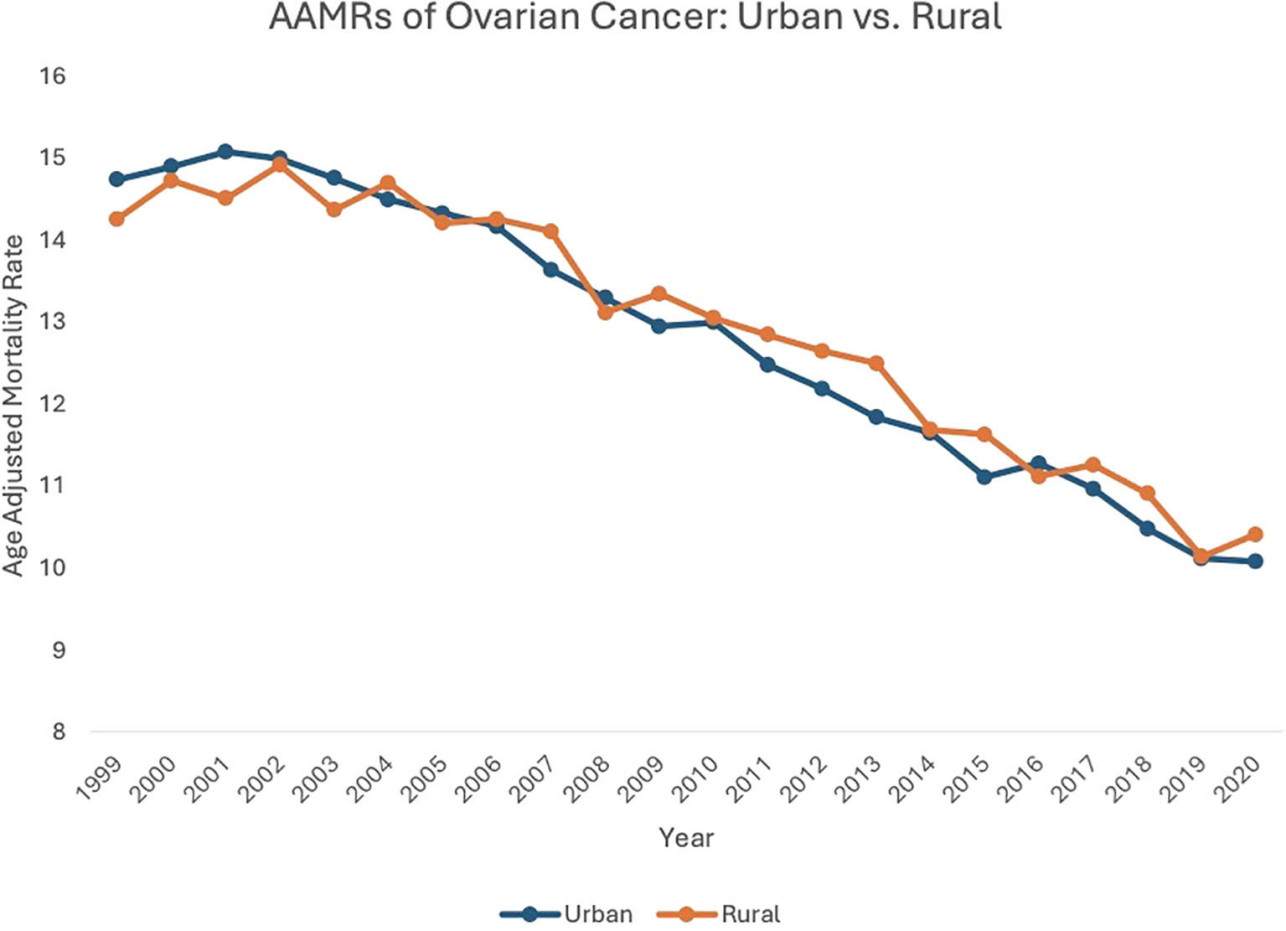
AAMRs of ovarian cancer: urban vs. rural.

Between 1999 and 2003, patients in urban settings saw an increase in AAMR, from 14.73 in 1999 to 14.75 in 2003 (APC: 0.27, 95% CI: -0.69 to 1.79). From 2003 to 2020, urban localities saw a subsequent decrease in AAMR, from 14.75in 2003 to 10.07 in 2020 (APC: -2.32, 95% CI: -2.45 to -2.20). Between 1999 and 2004, patients in rural areas saw an increase in AAMR, from 14.25 in 1999 to 14.69 in 2004 (APC: 0.51, 95% CI: -0.57 to 2.56), which then decreased to 10.40 in 2020 (APC: -2.20, 95% CI: -2.48 to -1.99) (**Figure 4**, **Supplementary Table 3**, **Supplementary Figure 4**).

### Ten-year age stratification

From 1999 to 2023, each ten-year age category analyzed saw a decrease in overall ovarian neoplasm-related mortality: 25–34 years old (AAPC: -0.33, 95% CI: -1.31 to 0.54); 35–44 years old (AAPC: -1.96, 95% CI: -2.24 to -1.71); 45–54 years old (AAPC: -2.09, 95% CI: -2.29 to -1.91); 55–64 years old (AAPC: -2.15, 95% CI: -2.39 to -1.89); 65–74 years old (AAPC: -2.05; 95% CI: -2.17 to -1.89); 75–84 years old (AAPC: -1.59; 95% CI: -1.76 to -1.34); 85+ years old (AAPC: -1.03, 95% CI: -1.34 to -0.75).

Between 1999 and 2013, patients 25–34 years old saw a decrease in AAMR, starting at 0.42 in 1999 decreasing to 0.32 in 2013 (APC: -1.76, 95% CI: -9.41 to -0.39), which then increased to 0.46 in 2023 (APC: 1.71, 95% CI: -0.39 to 11.00).

Between 1999 and 2023, patients aged 35–44 saw a decrease in AAMR, from 2.34 deaths per 100,000 people in 1999 to 1.48 in 2023 (APC: -1.96, 95% CI: -2.24 to -1.71). Patients aged 45–54 also saw an overall decrease in AAMR, from 8.73 in 1999 to 5.44 in 2023 (APC: -2.09, 95% CI: -2.29 to -1.91).

Patients aged 55–64 saw an overall decreasing trend in AAMR, from 20.88 in 1999 to 12.63 in 2023 (APC: -2.15, 95% CI: -2.39 to -1.89). Between 1999 and 2004, patients aged 65–74 saw an increase in AAMR, from 38.42 in 1999 to 38.71 in 2004 (APC: 0.27, 95% CI: -0.69 to 1.77), which then decreased to 23.07 in 2023 (APC: -2.65, 95% CI: -2.81 to -2.51).

Patients aged 75–84 saw an initial increase in AAMR, from 57.39 in 1999 to 60.52 in 2002 (APC: 1.74, 95% CI: -0.42 to 5.99). This AAMR then decreased to 38.59 in 2023 (APC: -2.05, 95% CI: -2.24 to -1.92).

**Figure 5:**
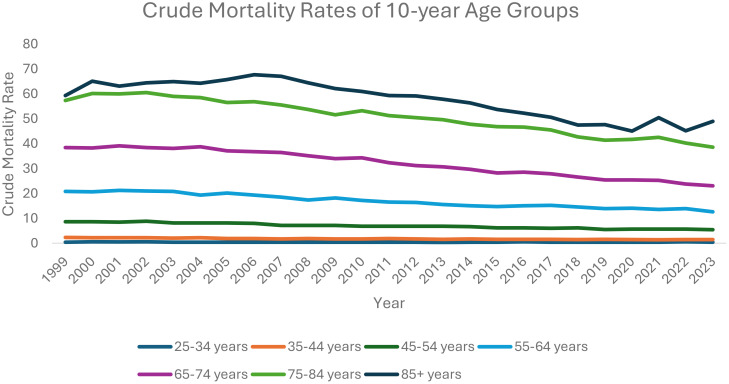
Crude mortality rates of 10-year age groups.

Between 1999 and 2007, patients aged 85+ saw an increase in AAMR, from 59.34 deaths per 100,000 people in 1999 to 67.09 in 2007 (APC: 1.01, 95% CI: 0.11 to 2.16). This AAMR then decreased to 45.05 in 2020 (APC: -2.81, 95% CI: -4.12 to -2.40) and subsequently increased to 48.99 in 2023 (APC: 1.41, 95% CI: -1.90 to 5.73) (**Figure 5**, **Supplementary Table 4**, **Supplementary Figure 5**).

### State level stratification

From 1999 to 2023, states such as Delaware, South Carolina, and Idaho experienced some of the larger decreases in AAMR amongst states. For instance, in Delaware, their AAMR decreased from 18.01 deaths per 100,000 people in 1999 to 9.83 in 2023. South Carolina’s AAMR decreased from 14.32 deaths per 100,000 people in 1999 to 8.22 in 2023. Idaho’s AAMR decreased from 17.90 deaths per 100,000 people in 1999 to 9.03 in 2023.

On the other hand, in the District of Columbia, AAMR increased from 16.20 deaths per 100,000 people in 1999 to 16.25 in 2023. From 2000 to 2023, the District of Columbia’s AAMR increased from 12.16 deaths per 100,000 people in 2000 to 16.25 in 2023. In the year 2020, the District of Columbia’s AAMR fell to 7.92 deaths per 100,000 people before increasing up to 16.25 in 2023 (**Supplementary Figures 6**-**9**, **Supplementary Table 5**). Changes in AAMR in each state throughout the study period can be found in **Supplementary Table 6**.

## Discussion

This study identified several key epidemiological trends and disparities in ovarian cancer mortality from 1999 to 2023, as previous studies have not captured the entirety of this time period in their data. First, racial differences in ovarian cancer mortality persist, but trends are complex. American Indian and Alaskan Native patients demonstrated a highly inconsistent, nonlinear trajectory while others demonstrated a steady decline in mortality. Second, substantial variability was observed at the state level despite a lack of variability in urban/rural results. While some states saw consistent and substantial decreases in age-adjusted mortality rates, others saw inconsistent improvement or even increased rates. Both urban and rural areas experienced declining mortality, although rural regions had higher levels of inconsistency. Lastly, mortality declined most sharply in middle-aged adults, particularly those aged 45-64, while women aged over 85 showed a recent increase in mortality rates due to ovarian cancer. It is interesting to note that the global burden of ovarian cancers increased from 1990 to 2021, with a positive correlation seen between cancer rates and the sociodemographic index (8). This underscores the complexity of ovarian cancer’s burden, both domestically and abroad, and highlights the importance of further research on this topic to improve overall ovarian cancer incidence and outcomes.

While prior studies have consistently reported racial disparities in ovarian cancer – particularly poorer survival and lower treatment rates among black women– our analysis tells a complex story. In terms of absolute mortality, Black or African American patients did not have disproportionately higher AAMRs as compared to other racial groups. In fact, their AAMRs were consistently lower than those of White patients and showed a consistent decline from 12.45 in 1999 to 8.73 in 2023. However, this trend should not be interpreted as evidence that disparities have been eliminated. Prior literature highlights that black women develop cancer at a younger age, have significantly lower 5 year mortality rates, and were less likely to receive guideline-recommended care (9–11) These disparities may not fully manifest in population-level mortality data but still remain critical to understanding inequities in ovarian cancer outcomes. Future studies are needed to assess whether the mortality improvements seen in Black and African American patients reflect genuine improvement in care or if there are confounding factors that are affecting these results.

American Indian and Alaskan native patients, however, demonstrated highly erratic and inconsistent trends. Notably, in 2015, this group had the highest AAMR of any racial group, only to fall to the second lowest in 2018. This inconsistency may reflect broader structural inequities, such as limited access to and availability of care but it also raises concerns about data quality. American Indian and Alaskan Native patients are known to be misclassified as other races, which may result in unrecognized disease burden and unstable trend reporting (12, 13). Research highlights the need for investment in healthcare resources for this population, as American Indian and Alaskan Native populations face poorer cancer outcomes and inadequate screenings (14) The instability in mortality trends among American Indian and Alaskan Native patients underscores the need for sustained investment in healthcare infrastructure within these communities. Programs such as the “walking forward program” currently deployed in South Dakota, show promise in decreasing stage-at-presentation and increasing treatment options available for American Indian and Alaskan Native patients (15).

It has been established that older patients (e.g., 70+) tend to be diagnosed with ovarian neoplasms at later stages of disease and likely have worse outcomes compared to younger patients (16). Our analysis seems to capture a similar picture when looking at ten-year age stratification with regards to AAMR. For instance, between 1999 and 2013, the AAMR of patients between 25–35 years old had declined, which then increased until 2023. However, we see a much higher AAMR in patients between 55–64 years old. While it is promising that each ten-year age category analyzed saw a decrease in overall ovarian neoplasm-related mortality, likely due to overall improvements in diagnostic and screening modalities and available treatment options, much more work and research need to be done to identify more effective diagnostic and treatment modalities to significantly reduce ovarian neoplasm mortality rates among older populations.

Geographic variability further complicates the national picture. While certain states, such as the District of Columbia, experienced increases in ovarian cancer mortality, other states, such as South Dakota, experienced steady declines in AAMRs throughout the study period. Notably, there was no apparent relationship between population distribution and mortality rates. This lack of correlation is further supported by the urban/rural analysis, which revealed consistent declines in AAMRs in both geographic settings (Rural and Urban). While certain states like Montana, New Hampshire, District of Columbia, Rhode Island, and West Virginia showed overall decreases in mortality from 1999 to 2023, they have recently started to display an increase in AAMRs. The District of Columbia displays the most drastic change, as their AAMR has more than doubled from 2020 to 2023. One possible explanation for this jump in AAMR is the COVID-19 pandemic. It has been previously established that overall cancer screenings and primary care visits declined during the COVID-19 pandemic (17, 18). Which means ovarian cancers that may have been detected and treated sooner without the pandemic were caught later and at a more aggressive stage – thus leading to increased mortality rates. However, further research is necessary to ascertain why only select states across the country with little association were directly affected by the increase in AAMR. It may be possible that certain healthcare policies before, during, and after this time period are influencing individual states’ statistics.

## Limitations

CDC WONDER, while offering useful public health data, may have some limitations. This study examined long term trends (1999-2023); thus it is important to recognize the fact that ICD coding or population standard changes over time could have influenced the comparability. For instance, while age, race, and geography certainly contribute to the social determinants of health that affect patient outcomes, many other factors that can impact population health are not adequately quantified by this database. For example, it is challenging to elucidate the impact of the COVID-19 pandemic on mortality rates, which could skew aspects of our analysis. Because the AAMRs calculated in this study utilize the US Census Bureau population estimates as denominators, some trends in AAMR could also be impacted by unavoidable variations in population counts. Additionally, barriers in access, affordability, and availability of care may not be adequately explained by geographic regions or race, which could be another potential limitation of our study.

## Conclusion

In conclusion, overall mortality rates due to ovarian neoplasms have decreased between 1999 and 2023. The results of this study, while only descriptive and hypothesis-driven rather than inferential, appear to highlight recent improvements in ovarian cancer mortality rates with regards to various demographic identifiers such as race, age, and geographic location. One possible explanation for this could be improvements in health disparities due to more robust public health efforts to address them. However, there are still many disparities that exist for patients of different age, race, and geographic groups that warrant further investigation in order to advance healthcare toward a more equitable future.

## Data Availability

The original contributions presented in the study are included in the article/**Supplementary Material**. Further inquiries can be directed to the corresponding author.

## References

[1] Ali M Tewari KS. A review of racial disparities in ovarian cancer and clinical trials. Curr Opin Obstetrics Gynecology. (2023) 36:23–7. doi: 10.1097/gco.0000000000000923, PMID: 38170549

[2] Sakhuja S Yun H Pisu M Akinyemiju T. Availability of healthcare resources and epithelial ovarian cancer stage of diagnosis and mortality among blacks and whites. J Ovarian Res. (2017) 10(1):57. doi: 10.1186/s13048-017-0352-1, PMID: 28830564 PMC5568254

[3] Chornokur G Amankwah EK Schildkraut JM Phelan CM. *Global ovarian cancer health disparities.* Gynecologic oncology (2013). Available online at: https://pmc.ncbi.nlm.nih.gov/articles/PMC3608795/. 10.1016/j.ygyno.2012.12.016 PMC3608795 23266352

[4] Mei S Chelmow D Gecsi K Barkley J Barrows E Brooks R. Health disparities in ovarian cancer. Obstetrics Gynecology. (2023) 142:196–210. doi: 10.1097/aog.0000000000005210, PMID: 37348095 PMC10278570

[5] Shah NS Lloyd-Jones DM O’Flaherty M Capewell S Kershaw KN Carnethon M. Trends in cardiometabolic mortality in the United States 1999-2017. JAMA. (2019) 322(8):780–2. doi: 10.1001/jama.2019.9161, PMID: 31454032 PMC6714016

[6] Surveillance Research Program, National Cancer Institute. *Joinpoint regression software*, version 5.4.0 (2025). Available online at: https://surveillance.cancer.gov/joinpoint (Accessed August 6, 2025).

[7] National Cancer Institute. Number of permuted data sets. In: Joinpoint help system (2025). Available online at: https://surveillance.cancer.gov/help/joinpoint/tech-help/frequently-asked-questions/numbere-of-permuted-data-sets (Accessed August 8, 2025).

[8] Li T Zhang H Lian M He Q Lv M Zhai L. Global status and attributab le risk factors of breast, cervical, ovarian, and uterine cancers from 1990 to 2021. J Hematol Oncol. (2025) 18(5). Available online at. doi: 10.1186/s13045-025-01660-y, PMID: 39794860 PMC11721161

[9] Howell EA Egorova N Hayes MP Wisnivesky J Franco R Bickell N. Racial disparities in the treatment of advanced epithelial ovarian cancer. Obstet. Gynecol. (2013) 122:1025–32. doi: 10.1097/AOG.0b013e3182a92011, PMID: 24104782 PMC3840948

[10] Park HK Ruterbusch JJ Cote ML. Recent trends in ovarian cancer incidence and relative survival in the United States by race/ethnicity and histologic subtypes. Cancer Epidemiol. Biomarkers Prev. (2017) 26:1511–8. doi: 10.1158/1055-9965.EPI-17-0290, PMID: 28751475 PMC6859937

[11] Liu FW Randall LM Tewari KS Bristow RE. Racial disparities and patterns of ovarian cancer surgical care in California. Gynecol. Oncol. (2014) 132:221–6. doi: 10.1016/j.ygyno.2013.08.035, PMID: 24016407 PMC4423747

[12] Agency for Healthcare Research and Quality. Racial Misclassification and Disparities in Mortality among AI/AN and Other Races (2014). Washington. Available online at: https://hcup-us.ahrq.gov/datainnovations/raceethnicitytoolkit/or26.jsp (Accessed August 8, 2025).

[13] Dankovchik J Hoopes M Nordstrom DL Knaster E. Racial misclassification and disparities in mortality among American Indians/Alaska Natives and other races. In: *Race and ethnicity data improvement toolkit.* Healthcare cost and utilization project (HCUP). Agency for Healthcare Research and Quality (2025). Available online at: https://hcup-us.ahrq.gov/datainnovations/raceethnicitytoolkit/or26.jsp (Accessed August 8, 2025).

[14] Guadagnolo BA Petereit DG Coleman CN. Cancer care access and outcomes for American Indian populations in the United States: Challenges and models for progress. Semin Radiat. Oncol. (2017) 27:143–9. doi: 10.1016/j.semradonc.2016.11.006, PMID: 28325240 PMC5363281

[15] University of South Dakota. Walking Forward Program strives to mend cancer disparities in American Indians (2023). South Dakotan M.D.: University of South Dakota Sanford School of Medicine. Available online at: https://www.usd.edu/academics/colleges-and-schools/sanford-school-of-medicine/south-dakotan-medicine/walking-forward-program-strives-to-mend-cancer-disparities-in-american-Indians (Accessed August 8, 2025).

[16] Mancebo G Sole-Sedeno JM Fabregó B Pinto G Vizoso A Alvarez M. Influence of age on treatment and prognosis in ovarian cancer patients. Cancers. (2025) 17:1397. doi: 10.3390/cancers17091397, PMID: 40361324 PMC12071169

[17] An LC Zarr-McDonagh A Krumm A Bacon E Liebrecht C Rennaker H. Initial and persistent changes in cancer screening in a US midwestern community health center network following the onset of COVID-19. Prev Med Rep. (2025) 53:103030. doi: 10.1016/j.pmedr.2025.103030, PMID: 40231219 PMC11994969

[18] Tu K Lapadula MC Apajee J Bonilla AO Baste V Cuba-Fuentes MS. Changes in reasons for visits to primary care after the start of the COVID-19 pandemic: An international comparative study by the International Consortium of Primary Care Big Data Researchers (INTRePID). PloS Glob Public Health. (2024) 4:e0003406. doi: 10.1371/journal.pgph.0003406, PMID: 39173045 PMC11341054

