# Supplementary material for: Overarching view of trends and disparities in malignant neoplasm of the ovary between 1999-2023: a comprehensive CDC WONDER database study
Source: Front Oncol. 2025 Nov 4;15:1691932. doi: 10.3389/fonc.2025.1691932 (PMC12623196; doi:10.3389/fonc.2025.1691932)
Supplement: Supplementary file 1 [file DataSheet1.docx]

|  | **Age Adjusted Mortality Rates** | | | | |
| --- | --- | --- | --- | --- | --- |
| Year | American Indian or Alaska Native | Asian or Pacific Islander | Black or African American | White | Hispanic |
| 1999 | 8.43 | 8.16 | 12.45 | 15.47 | 9.26 |
| 2000 | 7.35 | 8.06 | 12.23 | 15.77 | 10.56 |
| 2001 | 13.6 | 8.2 | 12.71 | 15.79 | 10.14 |
| 2002 | 9.33 | 8.62 | 12.76 | 15.91 | 9.99 |
| 2003 | 12.44 | 8.2 | 12.46 | 15.61 | 10 |
| 2004 | 10.48 | 7.71 | 12.84 | 15.35 | 10.04 |
| 2005 | 8.87 | 8.56 | 12.24 | 15.21 | 10.11 |
| 2006 | 12.62 | 8.04 | 11.65 | 15.13 | 10.19 |
| 2007 | 9.81 | 7.86 | 11.18 | 14.68 | 9.56 |
| 2008 | 10.69 | 8.44 | 11.15 | 14.11 | 9.37 |
| 2009 | 11.61 | 7.51 | 11.27 | 13.85 | 9.43 |
| 2010 | 11 | 7.96 | 11.26 | 13.79 | 9.21 |
| 2011 | 9.99 | 7.47 | 11.31 | 13.34 | 8.82 |
| 2012 | 11.06 | 7.32 | 11.16 | 13.02 | 9.46 |
| 2013 | 10.42 | 7.44 | 10.13 | 12.84 | 8.69 |
| 2014 | 8.36 | 6.92 | 10.76 | 12.4 | 8.59 |
| 2015 | 12.16 | 7 | 10.06 | 11.92 | 8.43 |
| 2016 | 9.96 | 8.02 | 9.64 | 11.98 | 8.15 |
| 2017 | 9.74 | 7.16 | 10 | 11.69 | 8.54 |
| 2018 | 7 | 6.96 | 9.66 | 11.21 | 8.09 |
| 2019 | 6.38 | 6.95 | 9.49 | 10.68 | 8.01 |
| 2020 | 9.74 | 7.38 | 9.38 | 10.71 | 7.6 |
| 2021 | 10.67 | 7.26 | 9.38 | 10.9 | 8.27 |
| 2022 | 7.41 | 7.29 | 8.85 | 10.44 | 8.13 |
| 2023 | 7.31 | 6.98 | 8.73 | 10.08 | 7.87 |
| **Number of Joinpoints (Years of Joinpoint)** | - | - | 1(2002) | 1(2003) | - |
| **APC Segment-1 (95% CI)** | - | - | -1.734*  (-3.74, -1.53) | -2.241*  (-2.36, -2.14) | - |
| **Average APC** | -1.186 | -0.747* | -1.369* | -1.785* | -1.231* |

**Supplementary Table 1.** Malignant Neoplasms of the Ovary mortality trends stratified by race, 1999-2023. APC with 95% confidence intervals show. p<0.05 indicates significance.

**Supplementary Figure 2. Multiple Joinpoint Models of Malignant Neoplasms of the Ovary by Race**

**
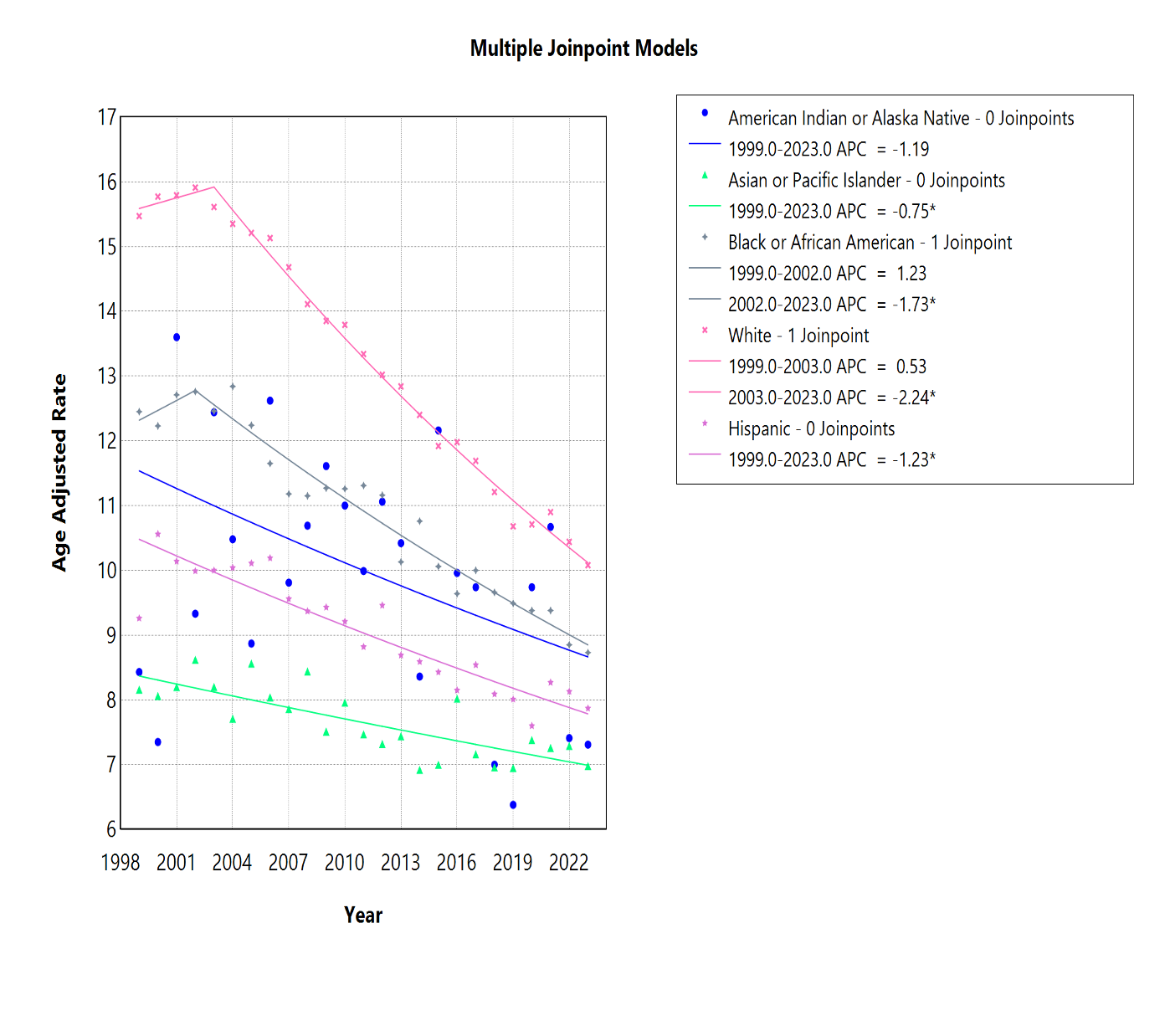
Supplementary Figure 2.** Multiple Jointpoint model of malignant neoplasms of the ovary by race. Overall and race stratified data regarding mortality rates related to malignant neoplasms of the ovary from 1999-2023.

|  | Age Adjusted Mortality Rates | | | |
| --- | --- | --- | --- | --- |
| Year | Northeast | Midwest | South | West |
| 1999 | 15.31 | 14.94 | 13.94 | 14.89 |
| 2000 | 15.35 | 15.04 | 14.25 | 15.36 |
| 2001 | 15.89 | 15.17 | 14.2 | 15.2 |
| 2002 | 15.58 | 15.44 | 14.34 | 15.13 |
| 2003 | 15.33 | 15.04 | 13.89 | 15.11 |
| 2004 | 14.63 | 14.65 | 13.9 | 15.36 |
| 2005 | 14.61 | 14.57 | 13.81 | 14.6 |
| 2006 | 14.97 | 14.59 | 13.64 | 13.93 |
| 2007 | 14.16 | 13.82 | 13.2 | 14.03 |
| 2008 | 13.79 | 13.41 | 12.86 | 13.32 |
| 2009 | 13.21 | 13.39 | 12.54 | 13.2 |
| 2010 | 13.19 | 13.34 | 12.6 | 13.05 |
| 2011 | 13.01 | 12.63 | 12.21 | 12.54 |
| 2012 | 12.5 | 12.44 | 11.93 | 12.51 |
| 2013 | 12.22 | 12.45 | 11.41 | 12.14 |
| 2014 | 11.83 | 11.79 | 11.18 | 12.1 |
| 2015 | 11.5 | 11.32 | 10.56 | 11.91 |
| 2016 | 11.19 | 11.66 | 10.76 | 11.76 |
| 2017 | 11.2 | 11.07 | 10.64 | 11.33 |
| 2018 | 10.35 | 10.33 | 10.48 | 10.89 |
| 2019 | 10.26 | 10.08 | 9.93 | 10.29 |
| 2020 | 10.46 | 10.21 | 9.83 | 10.22 |
| 2021 | 9.89 | 9.92 | 10.19 | 10.83 |
| 2022 | 9.73 | 9.72 | 9.64 | 10.44 |
| 2023 | 9.75 | 9.13 | 9.28 | 10 |
| **Number of Join points (Years of Joinpoint)** | 1 (2002) | 1 (2005) | 1(2004) | 1(2003) |
| **APC Segment 1(95% CI)** | -2.379*  (-2.54,-2.25 ) | -2.514*   (-2.81, -2.31) | -2.1344*  (-2.33, -1.99) | -2.078*  (-2.42, -1.93) |
| **Average APC** | -1.949* | -1.991* | -1.724* | -1.729* |

**Supplementary Table 2.** Malignant Neoplasms of the Ovary mortality trends stratified by region, 1999-2023. APC with 95% confidence intervals show. p<0.05 indicates significance

**Supplementary Figure 3. Multiple Joinpoint Models of Malignant Neoplasms of the Ovary by Census Region**

**
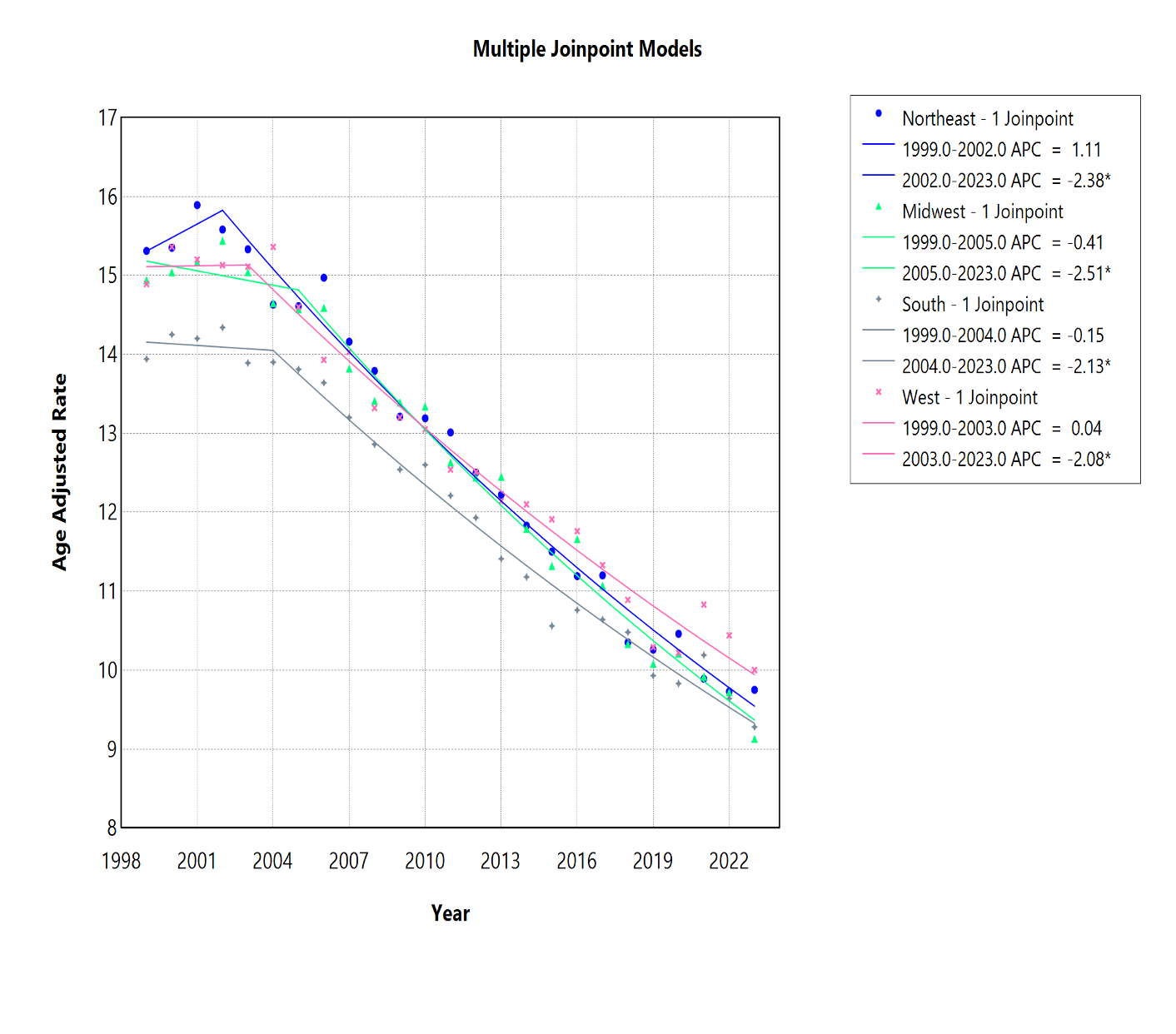
Supplementary Figure 3.** Multiple Jointpoint model of malignant neoplasms of the ovary by region. Overall and gender stratified data regarding mortality rates related to malignant neoplasms of the ovary from 1999-2023.

|  | Age Adjusted Mortality Rate | |
| --- | --- | --- |
| Year | Urban | Rural |
| 1999 | 14.73 | 14.25 |
| 2000 | 14.89 | 14.72 |
| 2001 | 15.07 | 14.5 |
| 2002 | 14.99 | 14.91 |
| 2003 | 14.75 | 14.36 |
| 2004 | 14.49 | 14.69 |
| 2005 | 14.32 | 14.2 |
| 2006 | 14.16 | 14.25 |
| 2007 | 13.63 | 14.1 |
| 2008 | 13.29 | 13.11 |
| 2009 | 12.94 | 13.34 |
| 2010 | 12.99 | 13.04 |
| 2011 | 12.47 | 12.84 |
| 2012 | 12.18 | 12.64 |
| 2013 | 11.83 | 12.49 |
| 2014 | 11.64 | 11.68 |
| 2015 | 11.1 | 11.62 |
| 2016 | 11.27 | 11.11 |
| 2017 | 10.96 | 11.25 |
| 2018 | 10.47 | 10.9 |
| 2019 | 10.11 | 10.13 |
| 2020 | 10.07 | 10.4 |
| **Number of Joinpoints (Years of Joinpoint)** | 1(2003) | 1(2004) |
| **APC Segment-1 (95% CI)** | -2.317*  (-2.45, -2.19) | -2.201*  (-2.47, -1.99) |
| **Average APC** | -1.828* | -1.562* |

**Supplementary Table 3.** Malignant Neoplasms of the Ovary mortality trends stratified by urban vs rural classification, 1999-2023. APC with 95% confidence intervals show. p<0.05 indicates significance.

**Supplementary Figure 4. Multiple Joinpoint Models of Malignant Neoplasms of the Ovary: Urban vs Rural Classification**

**
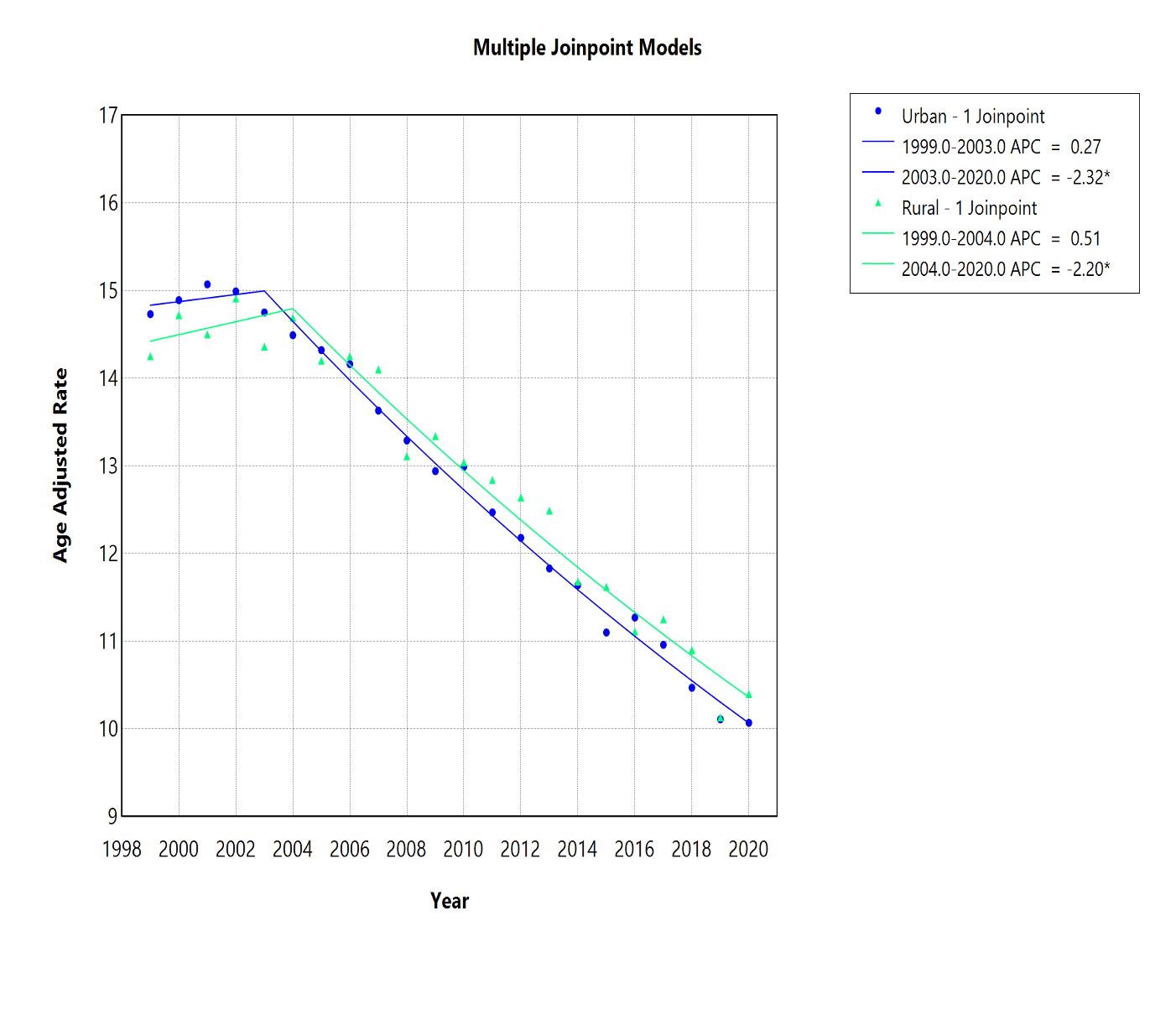
Supplementary Figure 4.** Multiple Jointpoint model of malignant neoplasms of the ovary by urban vs rural classification. Overall and race stratified data regarding mortality rates related to malignant neoplasms of the ovary from 1999-2023.

|  | **Crude Mortality Rate** | | | | | | |
| --- | --- | --- | --- | --- | --- | --- | --- |
| Year | 25-34 years | 35-44 years | 45-54 years | 55-64 years | 65-74 years | 75-84 years | 85+ years |
| 1999 | 0.42 | 2.34 | 8.73 | 20.88 | 38.42 | 57.39 | 59.34 |
| 2000 | 0.53 | 2.17 | 8.59 | 20.67 | 38.24 | 60.09 | 65.09 |
| 2001 | 0.54 | 2.2 | 8.48 | 21.23 | 39.13 | 59.94 | 63.18 |
| 2002 | 0.52 | 2.17 | 8.85 | 20.92 | 38.5 | 60.52 | 64.5 |
| 2003 | 0.47 | 2.09 | 8.18 | 20.79 | 38.14 | 59.08 | 64.96 |
| 2004 | 0.5 | 2.17 | 8.13 | 19.37 | 38.71 | 58.5 | 64.2 |
| 2005 | 0.45 | 1.98 | 8.14 | 20.17 | 37.16 | 56.53 | 65.8 |
| 2006 | 0.42 | 1.95 | 7.97 | 19.34 | 36.75 | 56.82 | 67.69 |
| 2007 | 0.42 | 1.73 | 7.23 | 18.44 | 36.4 | 55.52 | 67.09 |
| 2008 | 0.49 | 1.86 | 7.15 | 17.31 | 35.22 | 53.68 | 64.39 |
| 2009 | 0.45 | 1.7 | 7.15 | 18.19 | 33.92 | 51.62 | 62.17 |
| 2010 | 0.48 | 1.77 | 6.92 | 17.26 | 34.29 | 53.18 | 60.99 |
| 2011 | 0.42 | 1.84 | 6.89 | 16.62 | 32.4 | 51.27 | 59.38 |
| 2012 | 0.38 | 1.74 | 6.89 | 16.45 | 31.14 | 50.52 | 59.24 |
| 2013 | 0.32 | 1.63 | 6.82 | 15.51 | 30.69 | 49.57 | 57.89 |
| 2014 | 0.43 | 1.78 | 6.6 | 15.02 | 29.75 | 47.76 | 56.3 |
| 2015 | 0.44 | 1.59 | 6.15 | 14.79 | 28.15 | 46.84 | 53.76 |
| 2016 | 0.52 | 1.65 | 6.24 | 15.03 | 28.48 | 46.63 | 52.22 |
| 2017 | 0.4 | 1.51 | 6.04 | 15.26 | 27.92 | 45.54 | 50.61 |
| 2018 | 0.4 | 1.48 | 6.19 | 14.55 | 26.63 | 42.75 | 47.41 |
| 2019 | 0.42 | 1.5 | 5.5 | 13.95 | 25.38 | 41.41 | 47.72 |
| 2020 | 0.46 | 1.48 | 5.66 | 14.1 | 25.41 | 41.76 | 45.05 |
| 2021 | 0.46 | 1.49 | 5.66 | 13.62 | 25.34 | 42.53 | 50.5 |
| 2022 | 0.52 | 1.48 | 5.69 | 13.91 | 23.73 | 40.28 | 45.14 |
| 2023 | 0.46 | 1.48 | 5.44 | 12.63 | 23.07 | 38.59 | 48.99 |
| **Number of Joinpoints (Years of Joinpoint)** | 1(2013) | - | - | - | 1(2004) | 1(2002) | 2(2007,2020) |
| **APC Segment-1 (95% CI)** | 1.71  (-.039, 11.00) | - | - | - | -2.648*  (-2.81, -2.51) | -2.054*  (-2.24, -1.92) | -2.811* (-4.11, -2.39) |
| **APC Segment-2(95% CI)** | - | - | - | - | - | - | 1.413 (-1.90, 5.73) |
| **Average APC** | -0.328 | -1.962* | -2.091* | -2.150* | -2.046* | -1.587* | -1.027* |

**Supplementary Table 4.** Malignant Neoplasms of the Ovary mortality trends stratified by ten-year groups,1999-2023. APC with 95% confidence intervals show. p<0.05 indicates significance.

**Supplementary Figure 5. Multiple Joinpoint Models of Malignant Neoplasms of the Ovary by 10-Year Age Groups**


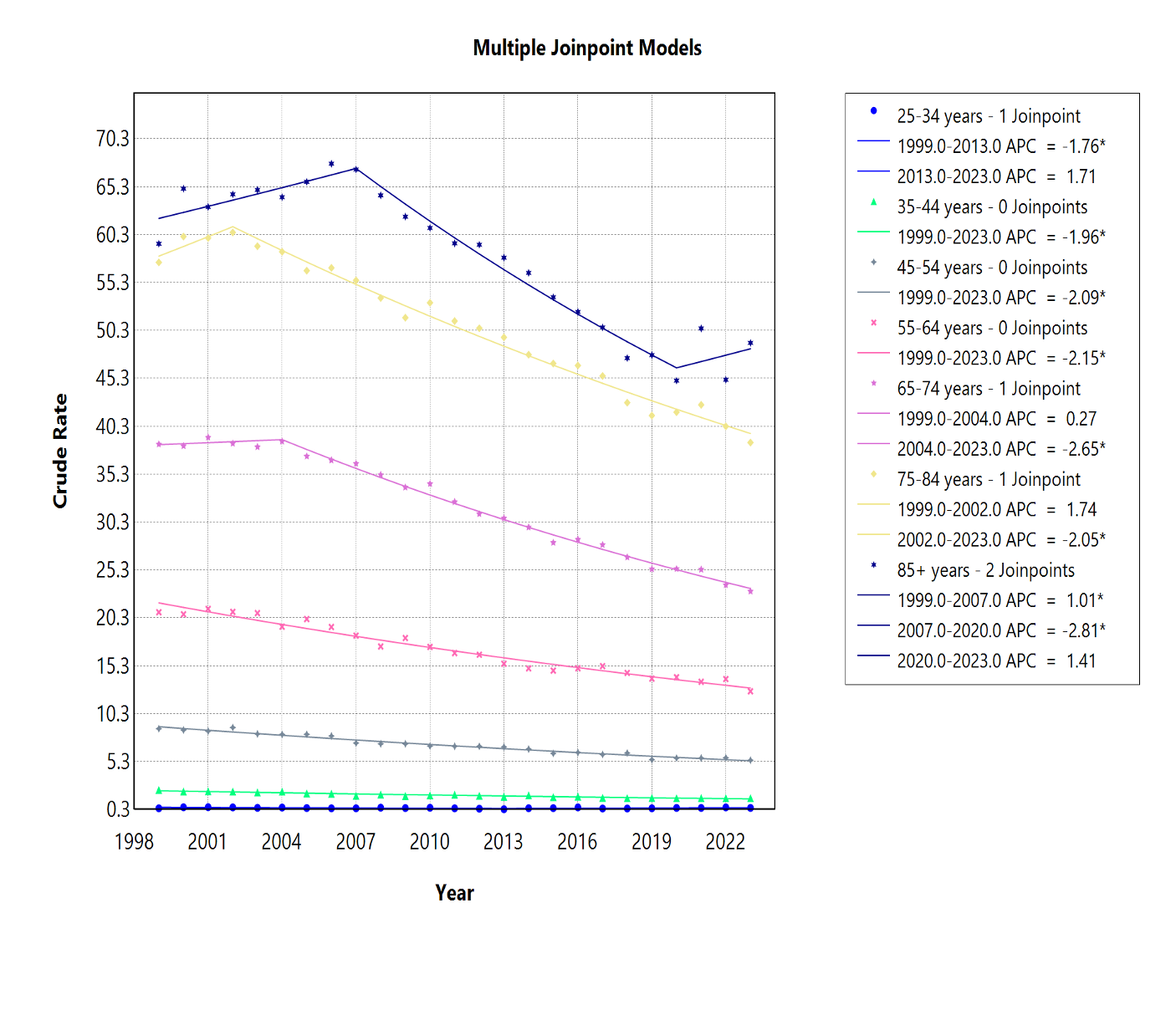
**Supplementary Figure 5.** Multiple Jointpoint model of malignant neoplasms of the ovary by ten-year groups. Overall and race stratified data regarding mortality rates related to malignant neoplasms of the ovary from 1999-2023.

| **Age Adjusted Mortality Rates per State** | | | | |
| --- | --- | --- | --- | --- |
| **State** | **1999** | **2000** | **2020** | **2023** |
| Alabama | 13.18 | 15.39 | 8.73 | 9.92 |
| Alaska | Unreliable | Unreliable | Unreliable | Unreliable |
| Arizona | 13.06 | 12.11 | 9.7 | 10.27 |
| Arkansas | 14.33 | 15.17 | 10.35 | 11.02 |
| California | 14.94 | 15.98 | 10.46 | 10.07 |
| Colorado | 14.69 | 14.56 | 10.87 | 9.45 |
| Connecticut | 15.14 | 14.03 | 10.25 | 8.77 |
| Delaware | 18.01 | 15.23 | 11.22 | 9.83 |
| District of Columbia | 16.2 | 12.16 | 7.92 | 16.25 |
| Florida | 13.72 | 14.06 | 9.65 | 8.64 |
| Georgia | 15.35 | 14.39 | 10.29 | 8.67 |
| Hawaii | 9.37 | 10.04 | 8.92 | 6.73 |
| Idaho | 17.9 | 15.69 | 9.78 | 9.03 |
| Illinois | 14.87 | 15.28 | 10.47 | 9.19 |
| Indiana | 16.17 | 15.94 | 10.15 | 8.97 |
| Iowa | 13.9 | 17.62 | 12.18 | 9.64 |
| Kansas | 14.5 | 15.96 | 10.61 | 9.51 |
| Kentucky | 14.87 | 13.83 | 8.89 | 8.95 |
| Louisiana | 12.93 | 13.27 | 9.9 | 8.29 |
| Maine | 16.96 | 18.45 | 10.72 | 8.95 |
| Maryland | 13.15 | 15.71 | 11.62 | 10.36 |
| Massachusetts | 14.49 | 14.72 | 10.33 | 10.38 |
| Michigan | 16.37 | 15.19 | 11.72 | 9.37 |
| Minnesota | 14.37 | 14.19 | 9.43 | 9.35 |
| Mississippi | 14.63 | 14.45 | 10.09 | 9.68 |
| Missouri | 15.22 | 13.64 | 9.38 | 8.84 |
| Montana | 16.41 | 15.73 | 8.8 | 12.29 |
| Nebraska | 13.05 | 16.13 | 10.39 | 8.41 |
| Nevada | 14.69 | 11.67 | 10.12 | 10.27 |
| New Hampshire | 15.96 | 13.87 | 8.15 | 12.14 |
| New Jersey | 16.2 | 16 | 10.31 | 9.61 |
| New Mexico | 11.3 | 14.33 | 9.34 | 9.02 |
| New York | 14.76 | 15.61 | 10.25 | 9.66 |
| North Carolina | 14.77 | 14.19 | 8.7 | 9.23 |
| North Dakota | 12.97 | 15.67 | 9.18 | 9.1 |
| Ohio | 14.32 | 14.01 | 9.67 | 8.74 |
| Oklahoma | 13.31 | 13.51 | 12.17 | 9.69 |
| Oregon | 16.87 | 16.81 | 10.36 | 9.58 |
| Pennsylvania | 15.95 | 15.46 | 11.45 | 9.73 |
| Rhode Island | 13.5 | 11.89 | 8.1 | 10.66 |
| South Carolina | 14.32 | 13.21 | 9.21 | 8.22 |
| South Dakota | 20.27 | 14.79 | 10.14 | 10.23 |
| Tennessee | 14.36 | 14.85 | 10.38 | 11.04 |
| Texas | 13.28 | 13.54 | 9.69 | 9.14 |
| Utah | 15.51 | 11.98 | 10.11 | 9.44 |
| Vermont | 12.04 | 12.33 | 10.89 | 10.23 |
| Virginia | 13.81 | 15.06 | 9.79 | 9.27 |
| Washington | 17.12 | 18.06 | 10.6 | 11.2 |
| West Virginia | 14.2 | 17.59 | 11.87 | 13.4 |
| Wisconsin | 13.85 | 15.39 | 9.03 | 9.34 |
| Wyoming | Unreliable | 15.46 | Unreliable | 11 |

**Supplementary Table 5.** Malignant Neoplasms of the Ovary Mortality trends stratified by state, 1999-2023.

**Supplementary Figure 6. National Trends of AAMRs of the Ovary, 1999-2020**


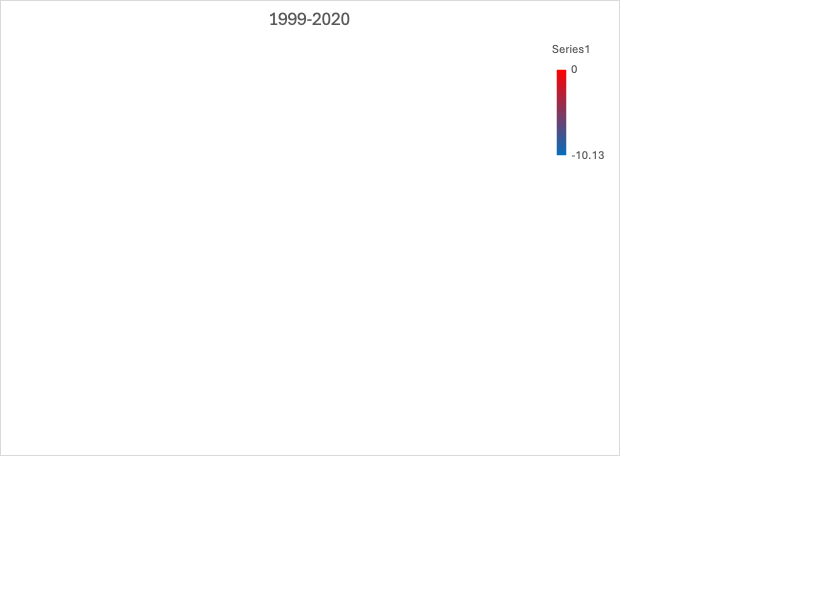


**Supplementary Figure 6.** National trends of Age Adjusted Mortality Rates (AAMRs) for Malignant neoplasms of the ovary, 1999 – 2020, based on CDC wonder data.

| **Age Adjusted Mortality Rate per State** | | | | |
| --- | --- | --- | --- | --- |
| **State** | **2023-1999** | **2023-2000** | **2020-1999** | **2020-2000** |
| Alabama | -3.26 | -5.47 | -5.47 | -6.66 |
| Alaska | #VALUE! | #VALUE! | #VALUE! | #VALUE! |
| Arizona | -2.79 | -1.84 | -1.84 | -2.41 |
| Arkansas | -3.31 | -4.15 | -4.15 | -4.82 |
| California | -4.87 | -5.91 | -5.91 | -5.52 |
| Colorado | -5.24 | -5.11 | -5.11 | -3.69 |
| Connecticut | -6.37 | -5.26 | -5.26 | -3.78 |
| Delaware | -8.18 | -5.4 | -5.4 | -4.01 |
| District of Columbia | 0.05 | 4.09 | 4.09 | -4.24 |
| Florida | -5.08 | -5.42 | -5.42 | -4.41 |
| Georgia | -6.68 | -5.72 | -5.72 | -4.1 |
| Hawaii | -2.64 | -3.31 | -3.31 | -1.12 |
| Idaho | -8.87 | -6.66 | -6.66 | -5.91 |
| Illinois | -5.68 | -6.09 | -6.09 | -4.81 |
| Indiana | -7.2 | -6.97 | -6.97 | -5.79 |
| Iowa | -4.26 | -7.98 | -7.98 | -5.44 |
| Kansas | -4.99 | -6.45 | -6.45 | -5.35 |
| Kentucky | -5.92 | -4.88 | -4.88 | -4.94 |
| Louisiana | -4.64 | -4.98 | -4.98 | -3.37 |
| Maine | -8.01 | -9.5 | -9.5 | -7.73 |
| Maryland | -2.79 | -5.35 | -5.35 | -4.09 |
| Massachusetts | -4.11 | -4.34 | -4.34 | -4.39 |
| Michigan | -7 | -5.82 | -5.82 | -3.47 |
| Minnesota | -5.02 | -4.84 | -4.84 | -4.76 |
| Mississippi | -4.95 | -4.77 | -4.77 | -4.36 |
| Missouri | -6.38 | -4.8 | -4.8 | -4.26 |
| Montana | -4.12 | -3.44 | -3.44 | -6.93 |
| Nebraska | -4.64 | -7.72 | -7.72 | -5.74 |
| Nevada | -4.42 | -1.4 | -1.4 | -1.55 |
| New Hampshire | -3.82 | -1.73 | -1.73 | -5.72 |
| New Jersey | -6.59 | -6.39 | -6.39 | -5.69 |
| New Mexico | -2.28 | -5.31 | -5.31 | -4.99 |
| New York | -5.1 | -5.95 | -5.95 | -5.36 |
| North Carolina | -5.54 | -4.96 | -4.96 | -5.49 |
| North Dakota | -3.87 | -6.57 | -6.57 | -6.49 |
| Ohio | -5.58 | -5.27 | -5.27 | -4.34 |
| Oklahoma | -3.62 | -3.82 | -3.82 | -1.34 |
| Oregon | -7.29 | -7.23 | -7.23 | -6.45 |
| Pennsylvania | -6.22 | -5.73 | -5.73 | -4.01 |
| Rhode Island | -2.84 | -1.23 | -1.23 | -3.79 |
| South Carolina | -6.1 | -4.99 | -4.99 | -4 |
| South Dakota | -10.04 | -4.56 | -4.56 | -4.65 |
| Tennessee | -3.32 | -3.81 | -3.81 | -4.47 |
| Texas | -4.14 | -4.4 | -4.4 | -3.85 |
| Utah | -6.07 | -2.54 | -2.54 | -1.87 |
| Vermont | -1.81 | -2.1 | -2.1 | -1.44 |
| Virginia | -4.54 | -5.79 | -5.79 | -5.27 |
| Washington | -5.92 | -6.86 | -6.86 | -7.46 |
| West Virginia | -0.8 | -4.19 | -4.19 | -5.72 |
| Wisconsin | -4.51 | -6.05 | -6.05 | -6.36 |
| Wyoming | #VALUE! | -4.46 | -4.46 | #VALUE! |

**Supplementary Table 6.** Malignant Neoplasms of the Ovary Mortality trends stratified by state, 1999-2023, showing differences between two different time frames.

**Supplementary Figure 7. National Trends of AAMRs of the Ovary, 2000-2020**


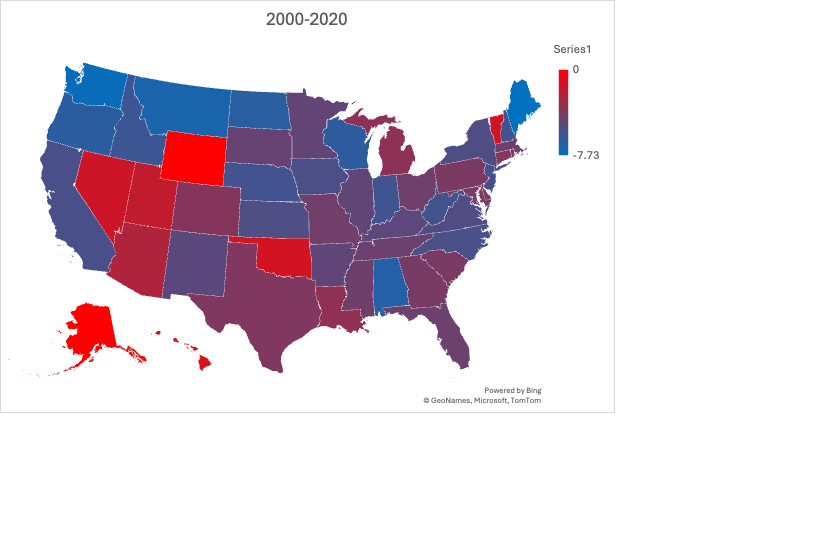


**Supplementary Figure 7.** National trends of Age Adjusted Mortality Rates (AAMRs) for Malignant neoplasms of the ovary, 2000 – 2020, based on CDC wonder data.

**Supplementary Figure 8. National Trends of AAMRs of the Ovary, 2000-2023**


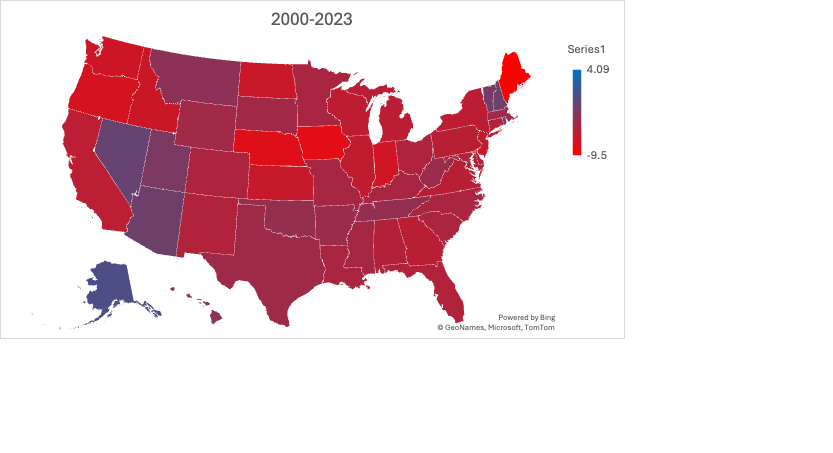


**Supplementary Figure 8.** National trends of Age Adjusted Mortality Rates (AAMRs) for Malignant neoplasms of the ovary, 2000 – 2023, based on CDC wonder data.

**Supplementary Figure 9 National Trends of AAMRs of the Ovary, 1999-2023**


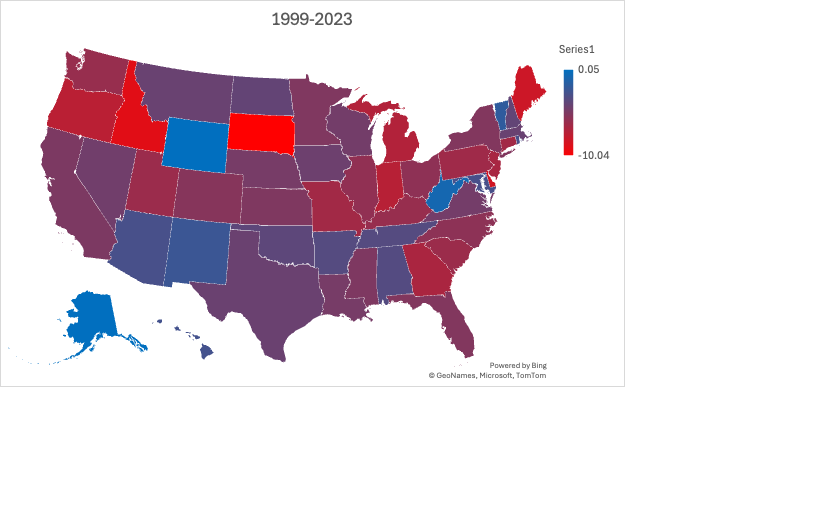


**Supplementary Figure 9.** National trends of Age Adjusted Mortality Rates (AAMRs) for Malignant neoplasms of the ovary, 1999 – 2023, based on CDC wonder data.
